# Surgical Treatment of Lung Cancer in Situs Inversus Totalis—A Case Report

**DOI:** 10.3390/reports6040046

**Published:** 2023-09-26

**Authors:** Janusz Wójcik, Tomasz Grodzki, Jarosław Pieróg, Norbert Wójcik, Dawid Kordykiewicz, Kajetan Kiełbowski, Maja Morozik, Stanisław Brożyna, Paulina Borowik, Małgorzata Edyta Wojtyś

**Affiliations:** Department of Thoracic Surgery and Transplantation, Pomeranian Medical University in Szczecin, Alfreda Sokołowskiego 11, 70-891 Szczecin, Poland; janusz.zenon.wojcik@wp.pl (J.W.); grodzki@grodzki.szczecin.pl (T.G.); jarpi@op.pl (J.P.); wojcik_norbert@wp.pl (N.W.); dawidkordykiewicz@onet.pl (D.K.); kajetan.kielbowski@onet.pl (K.K.); morozik1d@gmail.com (M.M.); stabro12@gmail.com (S.B.); paulina23116@gmail.com (P.B.)

**Keywords:** situs inversus totalis, lung cancer, thoracic surgery

## Abstract

Situs inversus totalis (SIT) is a congenital anomaly that involves the mirror rearrangement of the thoracic and abdominal internal organs. In this paper, we report a 56-year-old male patient with previously confirmed SIT, who was admitted to the hospital for the diagnosis and surgical treatment of non-small-cell lung cancer, located in the sixth right pulmonary segment. The patient underwent segmentectomy with mediastinal and hilar lymphadenectomy. Furthermore, we screened the PubMed and Embase databases for reports of the surgical treatment of patients with SIT and lung cancer. Articles describing inoperable and disseminated disease, as well as patients qualified for palliative treatment, were not included. Ultimately, we compared 21 articles (including the one described here). Data about the clinical condition, tumor characteristics, treatment, and histopathological examination were extracted and summarized.

## 1. Introduction

Situs inversus totalis (SIT) is a congenital anomaly that involves the mirror rearrangement of the thoracic and abdominal internal organs [1]. SIT is an uncommon abnormality, with its prevalence ranging from 1:6500 to 1:25,000 [2]. In contrast, lung cancer is one of the most common neoplastic diseases and is responsible for the highest cancer-related death rate [3]. Lung cancer in patients with SIT is extremely rare, mostly published in single case reports [4]. Moreover, the documentation of several such cases in one center is unique. The aim of this article is to report a third patient with SIT and lung cancer treated surgically in the authors’ center. Furthermore, there is an attempt to gather similar case reports published in the literature, and to describe diagnostic difficulties and management strategies for these patients.

## 2. Detailed Case Description

**Figure 1:**
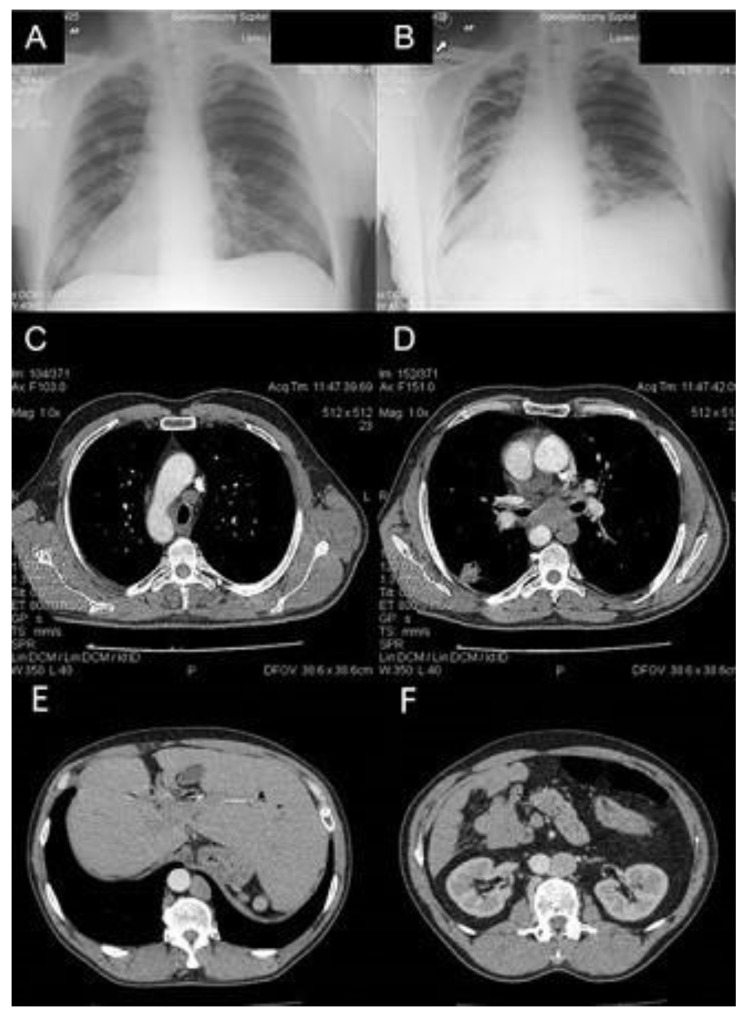
(**A**,**B**) Pre- and postoperative chest X-rays of the patient with SIT and lung cancer. (**C**,**D**) Chest CT showing rearrangement of mediastinal vessels and 2.5 cm tumor in VI right segment with enlargement of lymph nodes 4R and 7. (**E**,**F**) Abdominal CT showing position of the liver on the left side and position of the aorta on the right side.

A 56-year-old man with previously confirmed SIT was admitted to the Thoracic Surgery and Transplantation Department in January 2014 due to a 2.5 cm tumor of unknown etiology in the right lung, detected in a chest X-ray. The patient did not present any clinical symptoms. The image of SIT was confirmed in a contrast-enhanced chest computed tomography (CT). In addition, CT confirmed a 28 × 22 mm tumor in the sixth right segment, with a 9 mm satellite tumor located slightly higher, with enlargement of the mediastinal lymph nodes (Figure 1). Positron emission tomography FDG-PET/CT and endobronchial ultrasound (EBUS) were inaccessible during hospitalization. The diagnosis procedure was extended with an ultrasound fine needle aspiration biopsy of the right lung tumor and left supraclavicular lymph node with a diameter of 6 mm. Lung tumor biopsy was ineffective and cancer cells were not observed in the supraclavicular lymph node biopsy specimen, although the high level of carcinoembryonic antigen CEA (95.3 ng/mL) suggested adenocarcinoma in stage N2. A bronchofiberoscopy showed the right side of the lobar opening segmental bronchi anatomy in a configuration typical of the left lung, and an analogous image on the left side, where the bronchial configuration of the lobar openings’ segmental bronchi anatomy corresponded to the right lung. Subsequently, the patient qualified for surgery via right thoracotomy. Intraoperatively, the correct anatomy of the left lung (bilobar lung) on the right side and the tumor in the sixth segment, corresponding to the chest CT, was found; however, the posterior part of interlobar fissure was undeveloped. The entire anatomy of the mediastinum also corresponded to the left side, including the nodal station of the aorto-pulmonary window. Intraoperative examination confirmed metastases to the nodes of groups 5 and 7 and the procedure was limited to the sixth segmentectomy with mediastinal and lung hilar lymphadenectomy. The surgical technique was based on reconstructing the posterior part of the interlobar fissure by scissor stapling and ligation of the artery and vein to segment VI, followed by the linear stapler resection of segment VI.

Finally, the diagnosis of adenocarcinoma G2 and partially (approximately 20%) adenocarcinoma micropapillary G3 was established in the T1bN2Mx/IIIA stage. The postoperative course was uneventful. The patient underwent adjuvant chemo- and radiotherapy and died after 12 months due to metastatic brain spread.

## 3. Discussion

Situs anomalies are associated with the abnormal positioning of internal organs. Situs ambiguous refers to the improper position of one or several organs within the chest or abdomen (situs inversus incomplete syndrome). In contrast, situs inversus totalis (SIT) is a mirror rearrangement of the normal state (situs solitus) [1]. It is considered that the first reports of situs anomalies in humans were described in 17th century [5] Many studies have investigated the underlying genetics of SIT and found that several mutations might contribute to the development of this abnormality, including *CFAP45*, *NME7*, and *DNAH11*, among many others [6,7,8,9]. Patients with SIT or other laterality abnormalities often have an additional condition, named primary ciliary dyskinesia (PCD), which is associated with bronchiectasis. Indeed, SIT, bronchiectasis, and chronic sinusitis have collectively been described as Kartagener syndrome [10]. The occurrence of situs inversus in PCD patients is approximately 40% to 50% [11,12]. Pulmonary resection in a patient with Kartagener syndrome due to symptomatic bronchiectasis has also been described [13]. Patients with Kartagener syndrome may develop end-stage respiratory failure, which requires lung transplantation. Vascular and bronchial asymmetry between a recipient with situs inversus and a donor represents a technical challenge [14].

To the best of our knowledge, there are only 20 cases in medical databases that refer to patients with SIT and lung cancer treated surgically, with the operative verification of an inverted lung anatomy. The found cases with their characteristics are presented in Table 1.

In all the SIT and lung cancer operated cases, a mirror and typical anatomy were described, which supports the constant nature of the SIT anatomical rule, although there may be isolated changes in the number of segmental vessels, enlargement of the bronchial arteries with Kartagener syndrome cases, or massive infiltration requiring pneumonectomy [18,33]. This observation does not apply to situs inversus incomplete syndrome [35,36]. According to the mirror lung and mediastinum image, the largest challenge was the surgeon’s dexterity and reaction to the inverted anatomy. In our material, the layout of the operating team was typical. All maneuvers in the operational field were according to the rules for the opposing side. Segmental and main pulmonary vessels, lobar fissures, and bronchi based on a regular anatomy were supplied according to the technique used in the center (staplers, ligatures, electrocoagulation, etc.). Depending on the case, diagnostic procedures included bronchofiberoscopies, which visualized the reversed anatomy of the bronchial tree, and transbronchial and percutaneous transthoracic biopsies. Furthermore, surgery was often used for both diagnostic and curative purposes. Among the collected data, surgical procedures involved wedge resections (two patients), segmentectomies (five patients), lobectomies (twelve patients), bilobectomy (one patient), and pneumonectomy in two cases (one case had two operating methods). Open procedures (thoracotomy and median sternotomy) were performed in nine patients. A minimally invasive approach was used in the case of 12 patients, including one robotic-assisted procedure. Only eight studies reported a follow-up (6 months–2 years). The majority of cases were described in the eastern part of the world [15,18,19,21,22,24,25,26,28,29,30,31,33,34]. Surprisingly, the presence of as many as three such cases was observed in Szczecin, in Europe.

Lung cancer is one of the most common malignant diseases, but coexistence with SIT is not frequent. Coexisting lung cancer, SIT, and Kartagener syndrome is an even rarer observation [18,33]. The diagnosis of SIT is based on radiology. First, lung cancer and SIT cases are imaged only by X-ray [15]. Currently, radiological imaging based on X-ray, contrast-enhanced chest CT, and FDG-PET/CT is combined as 3D-CT, which allows the more accurate preoperative identification of the vascular and bronchial system [35]. The complex anatomy in SIT is a difficulty in the preoperative planning of thoracic procedures. Therefore, 3D-CT images were described in several included articles [28,29,33,34]. It is important to remember potential metastasis. PET/CT is an accurate method of determining such information, especially in non-small-cell lung cancer (NSCLC) patients, and a tissue biopsy should be performed even in uncommon metastasis locations [37].

The inverted but regular anatomy of SIT cases enabled the use of a typical surgical technique and even allowed the introduction of minimally invasive procedures. According to this observation, lobectomies and segmentectomies performed by the VATS technique have already been documented [38,39]. Robotic thoracic procedures are relatively novel and have gained much popularity recently. These procedures include early-stage lung cancer resections as well as complex procedures, such as sleeve resections [40,41]. Yang and colleagues recently published the first report of a lung cancer patient with SIT treated with robot-assisted lobectomy [28]. Furthermore, contemporary emphasis has been directed towards quantitative proteomics and its use in biomarker identification. MIC-A/B, FASLG, and HGF have gained prominence as indicators for the radical resection of NSCLC [42]. The combination of advanced surgical techniques with the development of biochemistry may be the future of lung cancer surgery.

## 4. Conclusions

To conclude, lung cancer may develop in SIT patients with a chance for surgery. Preoperative planning should be based on contrast-enhanced chest CT and FDG-PET/CT, combined with three-dimensional CT for the more accurate preoperative identification of the vascular and bronchial system. The CT mirror image has to be confirmed by bronchofiberoscopy. The analysis of the literature indicates that in cases of lung cancer with SIT, a normal, albeit inverted, lung anatomy can be counted on, which may be helpful in planning surgical treatment, especially in minimally invasive surgeries.

## Figures and Tables

**Table 1:** Characteristics of included patients. NR—not reported; VATS—video-assisted thoracoscopic surgery; RATS—robot-assisted thoracoscopic surgery.

Number	First Author	Sex	Age	Symptoms	Tumor Size	Tumor Location	TNM	Surgery	Type of Surgery	Pathology
1	Kodama [15]	male	68	chest pain and hemoptysis	80 × 70 mm	left lung (middle lobe and S7, S8)	NR	lobectomy	thoracotomy	squamous cell carcinoma
2	Subotich [16]	male	71	Productive cough, weight loss, and mild chest pain	NR	left lung (upper lobe)	NR	lobectomy	thoracotomy	adenocarcinoma
3	Bielewicz [17]	male	74	cough, weakness, fatigue	34 × 37 × 46 mm	left lung (middle and lower lobe)	pT2N0M0	bilobectomy	thoracotomy	squamous cell carcinoma
4	Inoue [18]	male	65	cough, hemoptysis	65 × 55 mm	left lung (upper lobe)	pT2bN1M0	pneumonectomy	median sternotomy	squamous cell carcinoma
5	Shimizu [19]	female	76	hemoptysis	48 × 32 × 20 mm	left lung (lower lobe)	pT4N1M0	lobectomy	thoracotomy	adenosquamous carcinoma
6	Wójcik [20]	male	69	cough, chest pain, hemoptysis	NR	left lung (upper lobe)	pT2aN0M1	pneumonectomy	thoracotomy	large cell carcinoma
7	Yoshida [21]	male	74	NR	21 × 10 × 15 mm	right lung (lower lobe)	pT2aN0M0	lobectomy	VATS	adenocarcinoma
8	Lin [22]	male	50	chest pain	60 × 50 × 50 mm	right lung (lower lobe)	pT4N0M0	lobectomy	thoracotomy	squamous cell carcinoma
9	Grapatsas [23]	male	80	NR	18 mm	left lung (lower lobe)	pT1aN0	lobectomy	thoracotomy	adenocarcinoma
10	Juma [24]	male	62	cough, chest pain, hemoptysis	32 × 31 mm	right lung (lower lobe)	pT2aN0M0	lobectomy	VATS	adenocarcinoma
11	Ye [25]	male	47	NR	25 mm	right lung (upper lobe)	pT2aN0M0	lobectomy	VATS	adenocarcinoma
12	Matsui [26]	male	68	NR	14 × 10 mm, 28 × 17 mm	right lung (upper and lower lobe)	pT1bN0M0	segmentectomy	VATS	squamous cell carcinoma
13	Gonzalez-Rivas [27]	female	48	NR	8 mm (S6) and 10 mm (S2)	right lung (upper and lower lobe)	NR	segmentectomy	VATS	minimally invasive adenocarcinoma (lower lobe) and adenocarcinoma in situ (upper lobe)
14	Kanayama [28]	female	61	NR	12 × 12 mm	left lung (upper lobe)	pT1bN0M0	lobectomy	VATS	adenocarcinoma
15	Wu [29]	female	41	asymptomatic	6 mm	left lung (upper lobe)	pTisN0M0	segmentectomy	VATS	adenocarcinoma in situ
16	Zhu [30]	female	58	fatigue, shortness of breath	16 × 14 mm	right lung (lower lobe)	pT1N0M0	lobectomy	VATS	lymphoepithelioma-like carcinoma
17	Chen [31]	female	68	NR	6 × 5 mm	left lung (lower lobe)	pTisN0M0	wedge	VATS	adenocarcinoma in situ
18	Celik [32]	female	50	COVID-19 pneumonia	15 × 14 mm	left lung (upper lobe)	pT1aN0M0	lobectomy	VATS	adenocarcinoma
19	Zhou [33]	female	74	no cancer-related symptoms	10 × 12 mm	left lung (upper lobe)	pTisN0M0	segmentectomy	VATS	adenocarcinoma in situ
20	Yang [34]	male	66	asymptomatic	38 mm, 8 mm	left lung (upper, middle lobe)	NR	lobectomy, middle wedge resection	RATS	adenocarcinoma, adenocarcinoma in situ
21	Presented Case	male	56	asymptomatic	27 × 20 × 15 mm	right lung (lower lobe)	T1bN2Mx	segmentectomy	thoracotomy	adenocarcinoma

## Data Availability

The data presented in this study are available in this article.
